# Impact of oral pathogens on inflammatory mediator expression in oral cells

**DOI:** 10.6026/973206300211506

**Published:** 2025-06-30

**Authors:** Pratik Sanjay Hodar, Mitesh Shah, Yihan Fu, Priti P. Shah, Abhishek Haldia, Anagha Agrawal, Miral Mehta

**Affiliations:** 1 Department of Prosthodontics & Crown and Bridge, Shri Yashwantrao Chavan Memorial Medical & Rural Development Foundation's Dental College & Hospital, Vadgaon Gupta Road, Ahmednagar - 414003, Maharashtra, India; 2 Department of Pathology, Bundelkhand Medical College, Sagar, Madhya Pradesh, India; 3 Department of General Dentistry, SmilebuilderzLancaster, PA United States; 4 Department of Oral Medicine and Radiology, Faculty of Dental Science, Dharmsinh Desai University, Nadiad, Gujarat, India; 5 Department of Pediatric and Preventive Dentistry, RUHS College of Dental Sciences, Jaipur, India; 6 Department of Public Health Dentistry, Narsinhbhai Patel Dental College and Hospital, Sankalchand Patel University, Visnagar, Gujarat, India; 7 Department of Pediatric and Preventive Dentistry, Karnavati School of Dentistry, Karnavati University, Gandhinagar, Gujarat, India

**Keywords:** Oral pathogens, inflammatory mediators, cytokines, *Porphyromonas gingivalis*, *Fusobacterium nucleatum*, *Aggregatibacter actinomycetemcomitans*, oral keratinocytes, periodontitis

## Abstract

Oral pathogens such as *P. gingivalis*, *F. nucleatum* and *A. actinomycetemcomitans* significantly elevate cytokine levels in oral epithelial cells. qRT - PCR analysis showed *P. gingivalis* induced a 4.2-fold increase in IL-1β, 3.8-fold in TNF-α, and 3.5-fold in IL-6 after 24 hours. The other pathogens also triggered a 2.4 to 3.6-fold cytokine rise (p < 0.05). This indicates their role in initiating oral inflammation through pro-inflammatory cytokine activation. Targeted microbial therapy may offer benefits in managing such conditions.

## Background:

The oral cavity maintains various microorganisms consisting of friendly and harmful species. Pathogenic oral bacteria play a significant role in developing inflammatory oral diseases like gingivitis and periodontitis although the microorganisms that live peaceably with the body assist with maintaining oral homeostasis [1, 2]. The periodontal tissue intruders *Porphyromonas gingivalis* together with *Aggregatibacter actinomycetemcomitans* and *Fusobacterium nucleatum* serve as leading microbial factors because they harm immune defenses while penetrating into gum tissues [3, 4]. Serious inflammatory responses occurs following pathogen-host interactions in the oral microbial environment by activating oral epithelial cells to produce IL-1β interleukin and IL-6 and TNF-α tumor necrosis factor-alpha [5, 6]. Bacteria in the oral cavity grow in the form of biofilms; these structures are subject to constant saliva or gingival crevicular fluid flow conditions [7]. The knowledge of how different pathogens drive the expression pattern of inflammatory mediators helps explains the fundamental processes behind oral inflammation development. The development of advanced *in vitro* models provides scientists with a better opportunity to accurately analyze interactions between oral cavity microorganisms and host cells. A controlled environment of cultured oral epithelial cells enables researchers to study cellular and molecular responses that develop from bacterial instigation. The models duplicate primary host defensive mechanisms especially when pathogens encounter epithelial tissue barriers at mucosal surfaces [8]. The study of microbial-stimulated changes in cytokine gene expression show individual species pathogenic potential to identify colonizing species from true periodontal pathogens. Because of their ability to manipulate host immune responses the three pathogens *P. gingivalis* and *F. nucleatum* and *A. actinomycetemcomitans* pose their main threat to periodontal health. The immune evading capabilities of *P. gingivalis* include its manipulation of Toll-like receptor signaling and *A. actinomycetemcomitans* creates harmful leukotoxins which target immune cells [9, 10]. The microorganism *F. nucleatum* helps create biofilms and enables the settlement of other microbial invaders by connecting early and late colonizers [11]. The pathogenic tactics employed by periodontal microbes lead to sustained inflammation and tissue damage because scientists now need to study their respective and compounded impact on inflammatory mediators. The study of molecular indicators linked to pathogen-initiated cytokine release provides valuable therapeutic approaches. Diagnostic methods making it possible to track bacteria which trigger excessive inflammation enable practitioners to design specific treatments involving antimicrobial medications as well as strategies to modulate the host response. Scientists investigate natural compounds together with antimicrobial peptides and nanoparticle-based drug delivery systems as treatment methods to minimize pathogen loads and tissue inflammations caused by cytokines [12, 13-14]. The examination of microorganisms with host response data progresses the development of individualized approaches to treat periodontal disease effectively. The understanding of how various oral pathogens influence cytokine expression in oral epithelial cells remains difficult to evaluate because existing research on this topic remains limited. Therefore, it is of interest to investigate the influence of specific periodontal bacteria on the gene expression levels of significant inflammatory mediators in laboratory-cultured oral epithelial cells.

## Materials and Methods:

## Cell culture:

A certified cell repository provided the Human oral keratinocyte cell line (HOK) which received nutrition from Dulbecco's Modified Eagle Medium (DMEM) containing 10% fetal bovine serum (FBS), 1% penicillin-streptomycin under 37°C humidified incubation with 5% CO^2^. The 6-well plates received HOK cell distribution at 1 x 10^6^ cells per well before cells achieved 80-90% confluence for treatment.

## Bacterial strains and preparation:

The oral pathogens *Porphyromonas gingivalis* (ATCC 33277), *Fusobacterium nucleatum* (ATCC 25586) and *Aggregatibacter actinomycetemcomitans* (ATCC 29523) were cultured under anaerobic conditions using standard procedures. Bacterial suspensions were prepared in phosphate-buffered saline (PBS) and concentrations were adjusted to 1 x 10^8^ CFU/mL using spectrophotometric methods.

## Cell stimulation:

Oral keratinocytes were exposed to each bacterial strain at a multiplicity of infection (MOI) of 100:1 for 24 hours. Uninfected cells served as negative controls. After incubation, cells were washed with PBS to remove non-adherent bacteria and harvested for RNA extraction.

## RNA isolation and cDNA synthesis:

Total RNA was extracted using a commercial RNA isolation kit according to the manufacturer's instructions. RNA purity and concentration were assessed using a spectrophotometer. Complementary DNA (cDNA) was synthesized from 1 µg of total RNA using a reverse transcription kit.

## Quantitative real-time PCR (qRT-PCR):

Expression levels of inflammatory cytokines IL-1β, IL-6 and TNF-α were quantified using qRT-PCR with SYBR Green chemistry. GAPDH was used as the housekeeping gene for normalization. Relative gene expression was calculated using the 2-ΔΔCt method. All reactions were performed in triplicate to ensure reproducibility.

## Statistical analysis:

Data were analyzed using GraphPad Prism software. Results were expressed as mean ± standard deviation (SD). One-way ANOVA followed by Tukey's post hoc test was used to determine statistical significance between groups. A p-value < 0.05 was considered statistically significant.

## Results:

Exposure of human oral keratinocytes to different oral pathogens resulted in significant upregulation of inflammatory cytokine gene expression when compared to the uninfected control group. As shown in Table 1 (see PDF), *Porphyromonas gingivalis* exposure induced the highest expression of IL-1β with a 4.2-fold increase, followed by *A. actinomycetemcomitans* (3.6-fold), and *F. nucleatum* (2.9-fold). Similar trends were observed in the expression levels of TNF-α and IL-6, where *P. gingivalis* led to 3.8- and 3.5-fold increases, respectively. The control group showed minimal cytokine expression, indicating a baseline level of inflammatory mediator activity in unstimulated cells. Statistical analysis using one-way ANOVA revealed significant differences between all test groups and the control group (p < 0.05). Tukey's post hoc test confirmed that the inflammatory response triggered by *P. gingivalis* was significantly higher than the responses induced by the other two pathogens. Table 1 (see PDF) illustrates the upregulation of inflammatory markers following bacterial exposure. These findings suggest that different oral pathogens have varied capacities to stimulate pro-inflammatory cytokine production in oral epithelial cells, with *P. gingivalis* being the most potent inducer among the tested organisms (Table 1 - see PDF).

## Discussion:

This research checked how three main oral pathogens-*Porphyromonas gingivalis*, *Fusobacterium nucleatum* and *Aggregatibacter actinomycetemcomitans*-would affect expression of critical inflammatory cytokines in oral keratinocytes. The experimental results showed that the three pathogenic bacteria significantly elevated IL-1β, TNF-α, and IL-6 production although *P. gingivalis* initiated the most pronounced inflammatory response. Previous studies have confirmed *P. gingivalis* status as a pathogenic pioneer that distorts host immunity to establish long-lasting inflammation [1, 2]. The pathogen contains virulence factors that include lipopolysaccharides (LPS) alongside gingipains and fimbriae which produce strong activation effects on Toll-like receptors while promoting significant cytokine creation [3, 4]. The research revealed that exposure to *P. gingivalis* produced a 4.2-fold boost in IL-1β expression levels while according to prior studies this bacterium effectively spurs IL-1β and other cytokine manufacture in periodontal tissue samples [5, 6]. Examine revealed *F. nucleatum* shows important cytokine expression levels especially through IL-6 enhances among the samples. The microorganism demonstrated prior behavior of binding to epithelial cells and modifying signaling pathways that involve NF-κB [7, 8]. The extent of cytokine stimulation by this microorganism strengthens its ability to support inflammatory processes however *P. gingivalis* maintains a superior role in such responses. *Aggregatibacter actinomycetemcomitans* which causes aggressive periodontitis triggered significant elevations of two major cytokines IL-1β and TNF-α.

Periodontal bacterium releases leukotoxin and cytolethal distending toxin that possibly activates host cells and induces pro-inflammatory immune activation [9, 10]. Laboratory research exists with comparable results showing *A. actinomycetemcomitans* induces inflammatory mediator elevation in epithelial cells and immune system cells [11, 12]. Periodontal inflammation develops and progresses through the elevated expression levels of IL-1β, IL-6, and TNF-α which were observed across all bacterial exposures [13]. Primary disease characteristics in periodontitis develop through cytokine actions which bring immune cells into the site while breaking matrix components and triggering bone tissue destruction [14]. Strategies that focus on blocking these bacterial species and their produced cytokines show potential to develop new treatment approaches for periodontal diseases [15]. This study has a limiting factor because its experimental design uses *in vitro* methods that cannot replicate the complete *in vivo* relationships between bacteria and tissue components during infection. Future research has to employ animal models or 3D tissue cultures to confirm these results that would simulate real human body conditions better.

## Conclusion:

Oral pathogens produce unique inflammatory responses in oral epithelial cells where *P. gingivalis* triggers the strongest reaction. Thus, we show the importance of microbial composition. They identify new targets which could serve as potential therapeutic points for managing periodontal disease outcomes.
